# Assessment of the Proximity of the Inferior Alveolar Canal with the Mandibular Root Apices and Cortical Plates—A Retrospective Cone Beam Computed Tomographic Analysis

**DOI:** 10.3390/jpm12111784

**Published:** 2022-10-28

**Authors:** Swati Srivastava, Hanan M. Alharbi, Afnan S. Alharbi, Mai Soliman, Elzahraa Eldwakhly, Manal M. Abdelhafeez

**Affiliations:** 1 Department of Conservative Dental Sciences, College of Dentistry, Qassim University, Buraydah 51452, Saudi Arabia; 2 General Dentistry, College of Dentistry, Qassim University, Buraydah 51452, Saudi Arabia; 3 Department of Clinical Dental Sciences, College of Dentistry, Princess Nourah Bint Abdulrahman University, P.O. Box 84428, Riyadh 11671, Saudi Arabia; 4 Faculty of Dentistry, October University for Modern Sciences and Arts, Giza 12451, Egypt

**Keywords:** cone beam computed tomography, cortical plates, endodontic mishaps, iatrogenic error, inferior alveolar canal, inferior alveolar nerve, mandibular teeth, neuropathy, over-instrumentation, paresthesia, proximity, root apex

## Abstract

Various endodontic interventions often lead to iatrogenic damage to the inferior alveolar nerve present in the inferior alveolar canal (IAC). The purpose of the present study was to analyze the relationships of IAC with the root apices of mandibular teeth and with the mandibular cortical plates. Materials: 116 cone beam computed tomography (CBCT) scans were examined and the shortest distance of IAC with the root apices of mandibular canines, premolars and molars, and with cortical plates was analyzed. The data were statistically analyzed using SPSS. Results: The shortest mean distance between IAC and lingual cortical plate (LCP) was found in the third molar area, and between IAC and buccal cortical plate (BCP) in the second premolar area. A high incidence of 60% direct communication (DC) was present in mandibular second molars; 38% in mandibular third molars; 13% in mandibular second premolars; 12% in mandibular first molars; and 1% in mandibular first premolars. Conclusion: Anteriorly, IAC was found to be significantly present in close approximation to the roots of mandibular canines. Posteriorly, IAC was found to be in significant proximity to the distal roots of mandibular second molars.

## 1. Introduction

The IAC originates from the mandibular foramen. It descends the ramus from the mandibular foramen mesially, and then it traverses horizontally in the body of the mandible just below the dental alveoli, or in close relation to the apices of the root and ends at the mental foramen. It consists of a neurovascular bundle that runs in the canal to branch into the mental nerve, which makes a loop and exits through the mental foramen near the apex of the second premolar or between the first and second premolars. The inferior alveolar nerve is enclosed within the IAC [1]. The literature shows that it has frequent anatomic variations and incorrect invasive procedures can lead to neuropathy in about 63% of cases [2]. Studies have also shown the close proximity of mandibular second and first molars with IAC [3,4]. Therefore, it is of paramount importance for a clinician to have knowledge of the various correlations that exist between the anatomical position of the apices of the root and the IAC. Variations in the positioning of the root apex relative to the IAC are an important risk factor to be considered while performing endodontic treatments. Such iatrogenic errors can lead to various neurologic deficits like anesthesia, paresthesia, and pain [5,6].

This close relationship of the IAC with the apices of mandibular teeth is of utmost importance when performing various endodontic procedures. In endodontics, the non-surgical procedures of both mandibular premolars and molars can lead to chemical or mechanical trauma. Procedures like over-instrumentation that mechanically traumatizes the nerve; pressure imposed by root canal sealers and endodontic points; and chemical trauma from root canal irrigants or root canal sealers can all lead to temporary or permanent damage of the neurovascular bundle, resulting in postoperative paresthesia or anesthesia. Moreover, gutta-percha products used as root canal filling materials are claimed to cause reactions ranging from none to chronic inflammation in soft and hard tissues. This happens due to the presence of oxides and antioxidants associated with the gutta-percha compound [7]. Thus, it is imperative that any extrusion from the root apices should be prevented. Numerous case reports have described anaesthesia of the lower lip, anaestheisa or parastheisa of gingiva, or anaestheisa or parastheisa of the mental nerve itself, clinically appearing immediately after root canal treatment [8,9,10]. The resulting anaestheisa or parastheisa of lips and gingiva usually decreases with time, but in some cases, it might cause irreversible anaestheisa or parastheisa of mental nerves [11]. It also adds to the possibility of reducing atypical fascial pain. Overfilling may cause persistent pain due to irritation of the overlying periosteum and mucosa [12]. Therefore, the presence of any of these materials in close proximity to IAC should be avoided. It is imperative for the clinician to understand the anatomical relationship of IAC with the mandibular teeth before performing any invasive endodontic procedure to avoid any inadvertent injury.

There are many radiographic techniques available to estimate the IAC position and its relationship to the surrounding structures. These include digital periapical radiographs, panoramic radiographs, spiral computed tomography, and cone beam computed tomographic scanning. Some of the former radiographic techniques have been used to locate the IAC, such as periapical radiography and panoramic radiography [1,13,14]. These imaging techniques have drawbacks such as superimposition of overlying anatomical structures, distortion, processing artifacts, acquisition presence, and inability to measure or analyze in three dimensions [15]. In recent times, CBCT has been used to assess the relationships of IAC with surrounding anatomical structures [16]. CBCT is a three-dimensional imaging modality that allows visualization of anatomical landmarks clearly without any superimpositions or distortion. It yields high resolution, no superimposition, without artifact, non-magnified and undistorted three-dimensional images of the maxillofacial anatomy that can be reformatted in any chosen plane for viewing and image management [17]. It is a relatively new imaging modality in dentistry that produces superimposition-free, high resolution, undistorted, artifact-free, and non-magnified three-dimensional images of maxillofacial structures that can be reformatted in any desired plane. This helps in interactive viewing and manipulation of the image. Moreover, the radiation dose of CBCT is significantly less as compared with conventional medical-grade computed tomography. Studies have shown CBCT to be consistent and accurate for measuring buccal and lingual cortical plate height and thickness. It is both a reliable and accurate method to obtain linear measurements for preoperative treatment planning [14].

Before any endodontic intervention, it is imperative for the clinician to be aware of the relative position of the IAC. The understanding of its anatomic variability and the course taken is helpful in treatment planning. As IAC is an important landmark and owing to the scarcity of research in this field in Saudi Arabia, we undertook this study. The present study was carried out to evaluate the relationships of the IAC with the root apices of mandibular teeth and the cortical plates of the mandible using cone-beam computed tomography images of the Saudi subpopulation.

## 2. Materials and Methods

### 2.1. Image Selection

The present retrospective study was conducted in the Department of Oral Medicine and Radiology. The Dental Research Ethics Committee (EA/6062/2021) of the institution granted approval for the study. All the CBCT scans were taken from an encrypted CBCT database. Furthermore, the patients were not exposed to unnecessary radiation for this research. A sample size calculation was performed using a 95% confidence interval so as to have a precision of 5%. CBCT scans of 116 patients in the age group of 18–75 years were analyzed in the study. The inclusion criteria were: scans containing the fully erupted mandibular permanent canines, premolars and molars, and a fully formed apex absent of any pathology. The exclusion criteria were: patients below the age of 18 years, presence of bone loss, pathology or congenital deformities of the mandible, poor quality CBCT scans, and previous endodontic surgery.

### 2.2. Imaging Analysis

Images were examined by using the scanner’s proprietary software (Sidexis XG 3D Viewer; Dentsply- Sirona Dental Systems, Montagematerial, Galileos, SK, Bensheim, Germany). The images were adjusted using the image processing tool in the software to ensure optimal visualization.

Patients were divided into different age groups as follows: 18–35 years, 36–55 years, and 56–75 years. The IAC was searched in the axial, coronal, and sagittal planes. The shortest distance was measured from the outer surface of the IAC to the following anatomic structures: LCP; BCP; inferior border of mandible and root apex of canine; first and second premolars; and mandibular molars. Orthogonal slices were used to measure the distance in millimeters, choosing the measuring tool on the 1:1 scale. The contrast and brightness of the images were adjusted to enhance the measuring procedure.

### 2.3. The Standard Consistency Test (Kappa Value)

All CBCT images were analyzed by two observers: an oral and maxillofacial radiologist and an endodontist (Srivastava S) with 10 years of experience. The observers were calibrated with the use of viewing software without any time restrictions to ensure inter-observer reliability. The images were analyzed to reach a consensus. In case of any disagreement, a third definitive evaluation was conducted by an endodontist (Abdelhafeez M) with 12 years of experience. The Kappa result was interpreted as per Cohen’s kappa coefficient: values ≤ 0 as indicating no agreement, 0.01–0.20 as none to slight, 0.21–0.40 as fair, 0.41–0.60 as moderate, 0.61–0.80 as substantial, and 0.81–1.00 as almost perfect agreement [18].

### 2.4. Statistical Analysis

The data were tabulated and subjected to statistical evaluation using Statistical Package for the Social Sciences (SPSS: an IBM company, version 22, Armonk, NY, USA). The categorical variables were assessed with the chi-square test. A *p* value of less than 0.05 was considered statistically significant, while a *p* value of more than 0.05 was considered statistically insignificant.

## 3. Results

An analysis of 116 scans was done, resulting in a total of 805 teeth. This included 21 canines, 173 first premolars, 121 premolars, 185 first molars, 207 molars, and 98 third molars.

**Figure 1:**
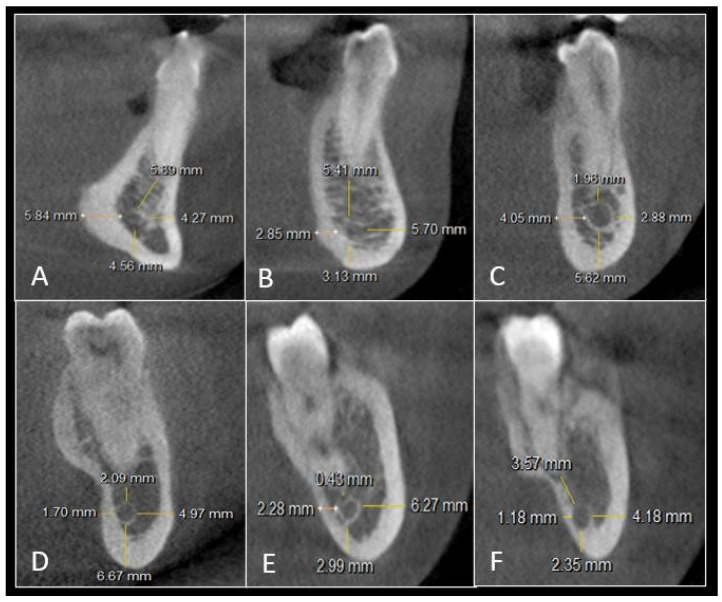
Cross-sectional view showing linear measurements of IAC with the root apex, BCP, inferior border of the mandible, and LCP in: (**A**) mandibular canine, (**B**) mandibular first premolar, (**C**) mandibular second premolar, (**D**) mandibular first molar, (**E**) mandibular second molar, and (**F**) mandibular third molar.

The shortest mean distance between IAC and LCP was found in the third molar area. The mean distance was 1.2 mm bilaterally. The total mean distance of IAC to LCP was shorter than the total mean distance of IAC from BCP except in the canine region. The shortest mean distance of 2.1 mm was noted on the right side and 2.6 mm on the left side in the region of the distal root of the mandibular third molar from IAC to the inferior border of the mandible. The shortest mean distance between IAC and BCP was found in the second premolar area. It was 3.7 mm on the right side and 3.6 mm on the left side. The canal coursed closer towards the LCP and the inferior border of the mandible as it ran posteriorly from the canine to the third molar. The apical position of the canal amongst all the teeth was shortest from the distal root of the second molar. The mean distance was 3.6 mm bilaterally. The IAC was found at the level of canine in 12 cases on the right side and 11 cases on the left side. It was placed more buccally. The mean distance from LCP was 5.6 mm on the right side and 5.4 mm on the left side. The mean distance between the IAC and the root apex of the canine was 3.5 mm bilaterally (Table 1) (Figure 1).

**Figure 2:**
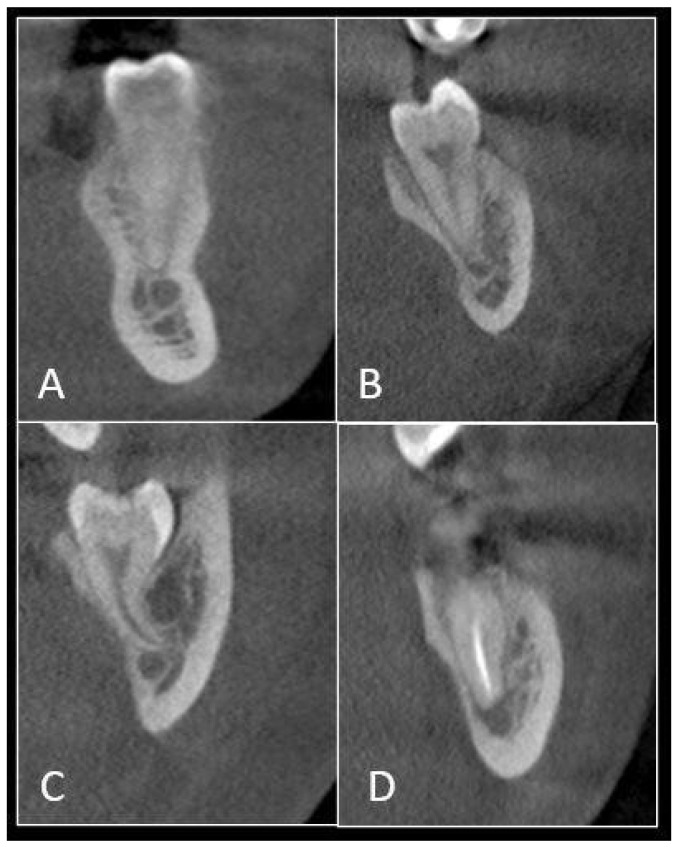
CBCT scan illustrating direct communication of the IAC with the root apex of: (**A**) mandibular premolar, (**B**) mandibular first molar, (**C**) mandibular second molar, and (**D**) mandibular third molar.

A high incidence of 60% was noted for mandibular second molars having DC of IAC with the apex of the roots. This was followed by 38% cases of mandibular third molar; 13% cases of mandibular second premolar; 12% cases of mandibular first molar; and 1% case of mandibular first premolar. No DC was seen for canines with the IAC (Table 2) (Figure 2).

The total mean distance between the IAC and the root apices of all teeth was 3.38 mm for the 18–35 age group; 4.35 mm for the 36–55 age group, and 4.46 mm for the 56–75 age group. The mean distance found in the 18–35 age group was significantly shorter than that found in the older age groups (Table 3).

Most of the cases showed symmetrical bilateral measurements, which were statistically not significant (*p* < 0.05). The results of the standard consistency check of the readings by the examiners was 0.81, indicating reliability of the results in the present study.

## 4. Discussion

The mandibular canal, also called the IAC, is a canal which encloses in it the inferior alveolar nerve, also called the inferior alveolar nerve, and blood vessels. It is typically located in close proximity to the root apices of mandibular teeth [19]. Following endodontic treatment of mandibular molars, lesions can occur commonly in the inferior alveolar nerve [20,21]. It can be due to root canal instrumentation during biomechanical preparation, where the instruments extend beyond the apical foramen to enter the IAC. It can also occur due to the forcing of root canal filling material into the IAC and inadequate debridement in the root canal [22,23]. Sometimes, inadvertent injections of root canal irrigants, especially sodium hypochlorite, beyond the apical foramen may cause inferior alveolar nerve injury, causing inevitable tissue necrosis [24]. Another potential complication of root canal treatment is the extrusion of infected debris from mandibular molars with apical periodontitis into the IAC. It has been stated previously that infected debris may destroy the perineurium of the inferior alveolar nerve, which is protective in nature. This leads to damage of nerve conductivity. After injury to the wall of the inferior alveolar nerve or injury to the IAC, a haematoma may develop leading to a rise in compression. This in turn can lead to complications like hypaesthesia, dysesthesia, hyperaesthesia, or complete anaesthesia [24,25]. Thus, understanding the proximity of the inferior alveolar nerve to the apices of mandibular teeth is imperative for the clinician before, during, and after the endodontic treatment is provided. Accurate knowledge will help in the prevention of iatrogenic errors.

A lot of anatomic variations are seen along the course of IAC, both in buccolingual and vertical dimensions. Dental clinicians should be aware of the distances between the IAC and the apices of the roots of mandibular teeth. They should also be familiar with the distance between IAC and the buccal cortical plate and lingual cortical plate. Inability to accurately assess and plan endodontic surgical or non-surgical treatment in this area leads to injury to the inferior alveolar nerve present within the IAC. Many studies have noted an increased risk of any kind of inadvertent injury during surgical or non-surgical endodontic procedures. According to Gerlach et al., 8.3% of sensory deficits are caused by damage to the IAN [26]. The injury to the inferior alveolar nerve might result in short-term or long-term altered sensation of the lower lip and chin. The management of such injuries can range from patient reassurance to surgical debridement depending on the severity of the damage and the speed with which corrective intervention is provided. Hence, it is mandatory for an astute clinician to have knowledge of the possible anatomical courses taken by IAC to prevent any mishaps.

In the present study, we analyzed CBCT scans anatomically. CBCT is a complex imaging modality in dentistry that has the ability to provide superior visualization of structures with respect to mandibular teeth, IAC, buccal cortical plate, and lingual cortical plate. It constructs high-resolution, superimposition-free, undistorted three-dimensional images of anatomic landmarks. It has been used to establish the course taken by IAC in many studies [27,28]. In CBCT, the IAC appears as a well-defined radiolucent zone surrounded by a radiopaque line. However, radiographically, the radiographic densities of the radiolucent and radiopaque borders are variable, and many cases have been reported with the radiographically invisible IAC [29].

The relationship of IAC with the apex of the mandibular roots is crucial in endodontics during treatment planning. Its close proximity with anterior teeth like the canine is significant. It entails a need for caution during endodontic surgeries in the canine-premolar area. In this study, we found the closest distance between the IAC to the root apex of the canine. In posterior teeth, the IAC was seen closest to the distal root of second molar. Our results are consistent with Shokry S.M. et al. (2019), who found the least distance between the second molar and IAC [29]. Aksoy et al. (2018) [30] also found that the distance between the second molar and IAC was significantly shorter as compared to premolars and first molars. Overall, the shortest mean distance of IAC from BCP was more than the shortest mean distance of IAC from LCP except in the region of canines. This indicated that the IAC was more lingually placed. Our results are in accordance with Tae et al. (2010), who also found a lingual course of IAC in the ramus and the body of the mandible [31].

Several studies have reported a closer proximity of IAC with the root apices of mandibular teeth, but very few studies have revealed a DC between the two. DC between the root apices is not rare and may be underestimated. In this study, the maximum incidence of DC was recorded in the mandibular second molar, followed by the mandibular third molar, and the mandibular first molar. Our findings are in accordance with Aksoy et al. (2018) who found a DC in 16% of cases of mandibular second molars and only 3.3% of mandibular first molars [30]. The least DC was found in mandibular premolars and no DC was found in mandibular canines.

Studies done by Yu et al. (2008) and Koivisto et al. (2016) showed that there was no statistically significant difference in change in location of IAC with respect to the apices of mandibular teeth in patients within the age group of 18–40 years [31,32]. However, this was in contradiction to our study. We found an increase in the mean distance between the IAC and the root apices of all teeth in different age groups. We found that the mean distance increased with the increase in age. The mean distance within the age group of 18–35 years was significantly shorter as compared to higher age groups. The result of this study indicates that as age increases, the distance between the IAC and the root apices of mandibular teeth increases. Kawashima et al. (2016) found that there is inferior migration of IAC with age due to increased bone growth after eruption of teeth [33]. Younger patients can have close proximity of IAC to the mandibular teeth, and this must be accounted for clinically when going for any invasive procedures. The vertical growth of the mandible is a continuous process of bone re-modeling [34]. The process of bone re-modeling and displacement of bone varies according to ethnicity, age, and sex [35]. Hence, these changes affect the location of IAC with respect to the mandibular molar root apices. In this study, the bilateral comparison of linear measurements was not statistically significant. These findings are different from those of Balaji et al. (2012), who found differences in bilateral comparison. This might be due to different ethnicities [36]. Our findings are in accordance with a study done by Vidya KC et al. (2019) and Yu et al. (2008) [31,37]. 

In recent years, clinical studies have pointed to the need to follow protocols with a proactive approach. These protocols help in maintaining a balanced oral microbiota. Scribante et al. (2022) have suggested the following protocols with a proactive approach while performing clinical practice [37]. The use of ozone therapy falls within the range of dental multidisciplinary. It has the ability to exert its antiseptic and antibacterial action. Ozone is divided into three oxygen molecules and thus has the ability to penetrate inside the DNA and RNA of bacteria and viruses, thereby destroying their structure. It is an assured way of reducing the time of healing by accelerating the healing action. This helps in lowering the incidence of both surgical and non-surgical endodontic oedema. Thus, a minimal invasive approach will keep the eubiosis constant in endodontic patients [38]. 

Clinical significance—This data can assist an astute clinician in the selection of modern files for minimal invasive endodontic instrumentation. They are specifically designed for this purpose, such as self-adjusting files (SAF, Redent Nova, Berlin, Germany), which allow for substance-preserving preparations; Hyflex CM (Coltene Whaledent, Cuyahoga Falls, OH, USA); ProTaper Gold (Dentsply, Maillefer, Ballaigues, Switzerland), and others, which employ advanced metallurgy to aid in minimal dentin destruction during root canal preparation.

Limitation of the study—This study was conducted on a small Saudi subpopulation. Future studies can target a larger sample size and a wider ethnicity to determine the outcomes of this study.

## 5. Conclusions

This study found a close proximity between the IAC and the mandibular canine anteriorly, and it was closest to the distal root of the mandibular second molar posteriorly. The study has also highlighted the DC of IAC with the roots of mandibular second molars and closer proximity with the root apices of the mandibular teeth in younger age groups. Identifying the variations of anatomical courses taken by IAC may help in pre-endodontic assessment and treatment planning of surgical or non-surgical endodontic treatment of mandibular teeth. Three-dimensional analysis with the help of CBCT provides exact images which are free of superimposition and distortions of the relevant anatomic structures. This will facilitate a clinician in achieving a favorable endodontic outcome.

## Figures and Tables

**Table 1:** Shortest mean distance (mm) from the IAC to the LCP, BCP, inferior border of the mandible, and root apex bilaterally.

Teeth		Right Side				Left Side			
		LCP	BCP	Inferior Border of Mandible	Root Apex	LCP	BCP	Inferior Border of Mandible	Root Apex
Canine		5.6	4.8	8.9	3.5	5.4	4.9	9.1	3.5
PM-1		3.4	4.5	8.7	4.7	3.4	4.3	8.7	4.6
PM-2		3.1	3.7	8.4	4.4	3.4	3.6	8.4	4.1
M-1	Mesial root	2.9	4.9	7.6	4.4	2.9	5.2	7.4	4.1
	Distal root	2.6	4.7	7.2	4.8	2.7	4.6	7.5	4.6
M-2	Mesial root	2.5	5.9	6.2	4.2	2.2	5.7	5.9	4.1
	Distal root	2.4	5.4	6.4	3.6	2.1	5.8	6.1	3.6
M-3	Mesial root	1.8	4.3	2.4	3.8	1.7	4.7	2.7	3.7
	Distal root	1.2	4.1	2.1	3.9	1.2	3.9	2.6	3.8

**Table 2:** Number (n) and percentage (%) of cases with DC of IAC to the apex of the root.

	Total No. of Teeth	DC n (%)
Canine	21	0
PM-1	173	1
PM-2	121	13
M1	185	12
M2	207	60
M3	98	38

**Table 3:** Mean distance (mm) between the IAC and the root apices of all teeth in various age groups. A different superscript uppercase in the same column indicates a statistically significant difference (*p* < 0.05).

Age	n	Mean Distance (mm)
18–35	161	3.38 ^a^
36–55	431	4.35 ^b^
56–75	213	4.46 ^b^

(n = number of teeth in each age group).

## Data Availability

The data presented in this study are available on request from the corresponding author.
